# A CT-based radiomics nomogram for the differentiation of pulmonary cystic echinococcosis from pulmonary abscess

**DOI:** 10.1007/s00436-022-07663-9

**Published:** 2022-10-01

**Authors:** Yan Li, Yaohui Yu, Qian Liu, Haicheng Qi, Shan Li, Juan Xin, Yan Xing

**Affiliations:** 1 School of Basic Medical Sciences, Xinjiang Medical University, Urumqi, Xinjiang China; 2 Imaging Center, The First Affiliated Hospital of Xinjiang Medical University, No 137, LiYuShan South Road, Urumqi, 830011 Xinjiang China; 3 State Key Laboratory of Pathogenesis, Prevention and Treatment of High Incidence Diseases in Central Asia, Medical Imaging Center, The First Affiliated Hospital of Xinjiang Medical University, Urumqi, Xinjiang China

**Keywords:** Radiomics, Nomogram, Pulmonary cystic echinococcosis, Pulmonary abscess, Computed tomography

## Abstract

The purpose of this study was to establish a clinical prediction model for the differential diagnosis of pulmonary cystic echinococcosis (CE) and pulmonary abscess according to computed tomography (CT)-based radiomics signatures and clinical indicators. This is a retrospective single-centre study. A total of 117 patients, including 53 with pulmonary CE and 64 with pulmonary abscess, were included in our study and were randomly divided into a training set (*n* = 95) and validation set (*n* = 22). Radiomics features were extracted from CT images, a radiomics signature was constructed, and clinical indicators were evaluated to establish a clinical prediction model. Finally, a model combining imaging radiomics features and clinical indicators was constructed. The performance of the nomogram, radiomics signature and clinical prediction model was evaluated and validated with the training and test datasets, and then the three models were compared. The radiomics signature of this study was established by 25 features, and the radiomics nomogram was constructed by using clinical factors and the radiomics signature. Finally, the areas under the receiver operating characteristic curve (AUCs) for the training set and test set were 0.970 and 0.983, respectively. Decision curve analysis showed that the radiologic nomogram was better than the clinical prediction model and individual radiologic characteristic model in differentiating pulmonary CE from pulmonary abscess. The radiological nomogram and models based on clinical factors and individual radiomics features can distinguish pulmonary CE from pulmonary abscess and will be of great help to clinical diagnoses in the future.

**Supplementary Information:**

The online version contains supplementary material available at 10.1007/s00436-022-07663-9.

## Introduction

Echinococcosis is an important parasitic zoonotic disease throughout the world. Two main species of medical and public health importance are *Echinococcus granulosus* (*E. granulosus*) and *Echinococcus multilocularis* (*E. multilocularis*), both of which can cause cystic echinococcosis (CE) and alveolar echinococcosis (AE) (Woolsey and Miller, 2021; Ravis et al. 2018). These parasites can cause disease in both humans and animals, resulting in serious health and economic problems (Deplazes et al. 2017). The WHO has placed CE on a list of 7 neglected zoonotic diseases that require priority intervention (Bonelli et al. 2021). Areas of Western China including Xinjiang, Tibet, Qinghai, Sichuan, Gansu and Ningxia have among the highest incidences of CE. Echinococcosis can affect many organs in the human body, particularly the lungs, which are the second most common organ affected (CAI et al. 2020; Wen et al. 2019).

**Fig. 1:**
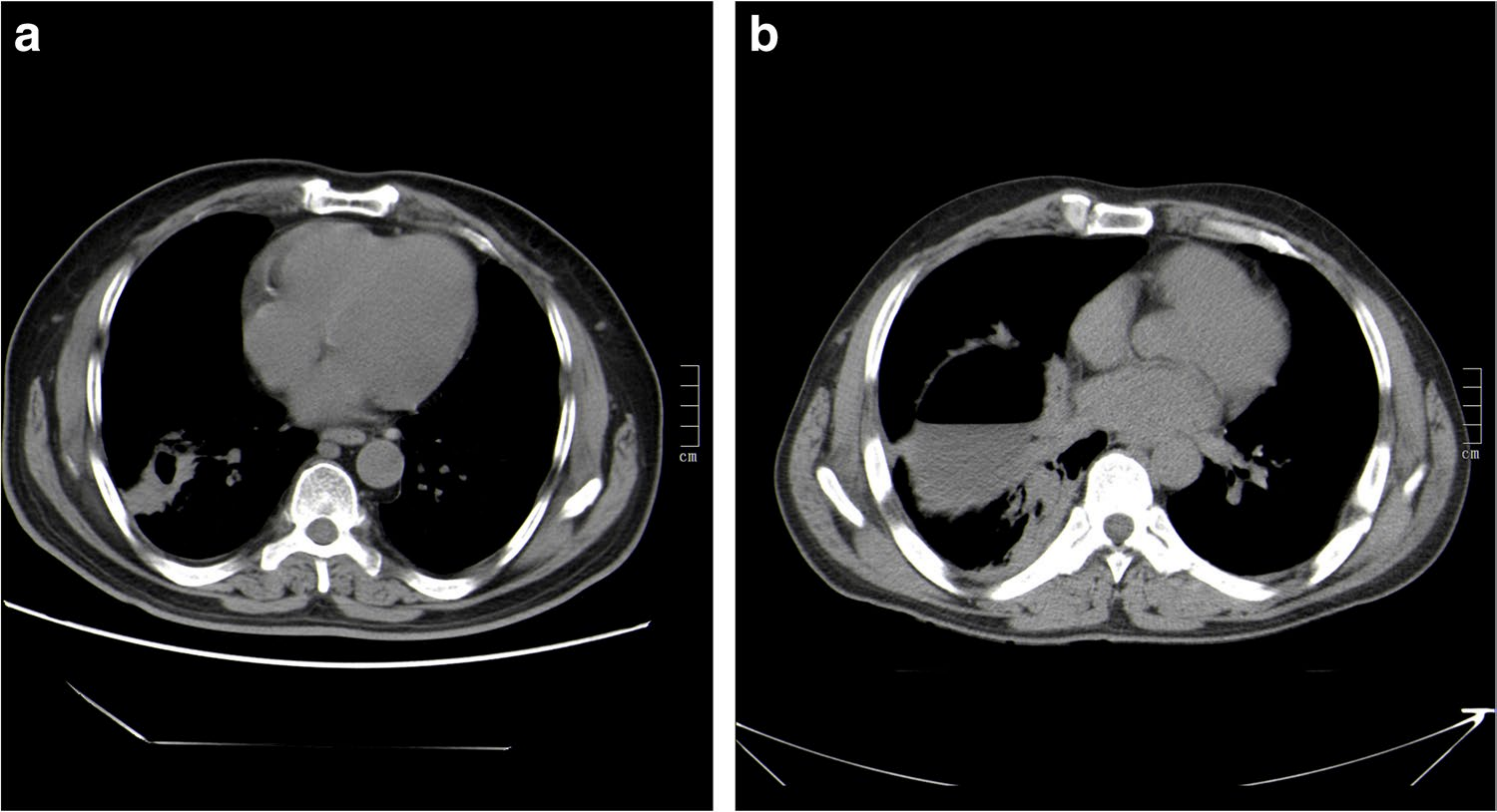
CT images of **a** pulmonary CE and **b** pulmonary abscess

The diagnosis of pulmonary CE is particularly challenging because it can have nonspecific symptoms and signs (Taxy et al. 2017). The early symptoms of the disease are usually not obvious, and the late symptoms do great harm to human health. Multislice computed tomography (MSCT) is the first choice for the clinical diagnosis of pulmonary CE (Wu et al. 2021; Giri and Parija 2012). If the cysts in pulmonary CE have not ruptured, the diagnosis is not very difficult, and the cysts can appear clearly as a round-like hypodense lesion with smooth edges on CT. However, the rupture rate of pulmonary CE is higher than that of hepatic CE (Hamouri et al. 2021). The typical imaging characteristics of pulmonary CE disappear after rupture and infection and need to be differentiated from those of pulmonary abscess (Morar and Feldman 2003). In clinical practice, it is difficult to distinguish pulmonary CE from pulmonary abscess with imaging alone (Fig. 1a, b). If misdiagnosed, secondary infections and severe allergic reactions can occur. When the lesion is punctured and drained, it will also cause hydatid fluid and hydatid sand protoscoleces to leak and spread, which will affect the human body, causing greater harm (Ye et al. 2016; Mohammed et al. 2021). Therefore, it is very important to improve the differential diagnosis of pulmonary CE and pulmonary abscess.

Radiomics uses high-throughput calculations to quickly extract a large number of quantitative image features from tomographic images (MRI, CT, PET) and to convert them into explorable, high-fidelity digital data. Through quantitative analysis of imaging data, various pathophysiological processes and their relationships can be analysed (Limkin et al. 2017; Chen et al. 2017, 2020; Aerts et al. 2014). Radiomics has been applied to the identification of chest diseases and prognostic evaluation, providing a new method for the differential diagnosis of pulmonary CE. In this study, we sought to develop and validate a radiomics nomogram that incorporates a radiomics signature and clinical factors for the differentiation of pulmonary CE and pulmonary abscess.

## Materials and methods

### Patients

This retrospective study was approved by the Ethics Committee of the First Affiliated Hospital of Xinjiang Medical University, and all methods were implemented in accordance with the Declaration of Helsinki and relevant guidelines and regulations. Due to the retrospective nature of the study, a waiver for informed consent was approved by the Ethics Committee of First Affiliated Hospital of Xinjiang Medical University. Consecutive patients were identified by searching the pathology database of our institution for the period from January 2010 to January 2020. A total of 117 patients with a diagnosis of pulmonary CE (*n* = 53, 18 men and 28 women) or pulmonary abscess (*n* = 64, 26 men and 29 women) were enrolled in this study. The inclusion criteria were as follows: (1) lesions identified via pathological results or clinical confirmation; (2) pulmonary CE complicated with rupture infection; and (3) complete clinical information. The exclusion criteria were as follows: (1) CE with no or multiple ruptures on CT images; (2) CT images with motion artefacts, poor image quality, different scanning conditions and inconsistent slice thickness; and (3) incomplete clinical information or loss to follow-up.

**Fig. 2:**
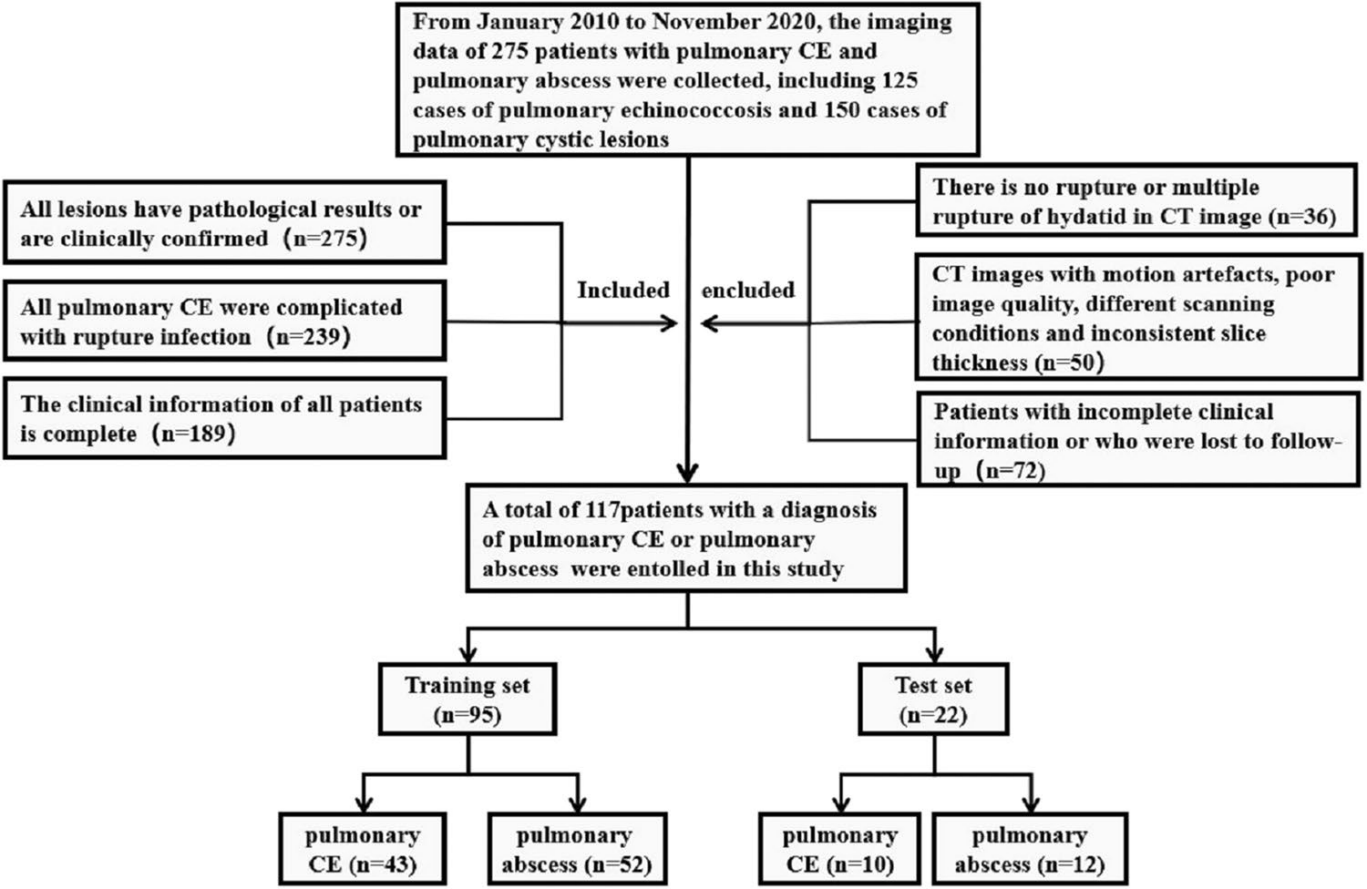
Flowchart for the selection of the study population

The electronic medical data of the patients were retrieved from the hospital information system. High-resolution computed tomography images were retrieved from the hospital image archiving and communication system. Laboratory examination results were retrieved from electronic medical records in the hospital information system. Baseline clinical data, including patient age, sex and laboratory indicators, including white blood cell (WBC) (i.e. leukocyte), neutrophil, monocyte, lymphocyte, eosinophil, interleukin-6 (IL-6), fibrinogen (FIB), high-sensitivity C-reactive protein (HS-CRP) and plasma D-dimer levels, were also analysed by reviewing the medical records and serial CT imaging data. The location, density and size of lesions on CT images and patient body temperature and sputum were also analysed. All patients were randomly allocated to the training or test set at a ratio of 8:2, which resulted in 95 patients being allocated to the training set and 22 patients being allocated to the test set. A flowchart for the selection of the study population is shown in Fig. 2.

### CT image acquisition

All patients underwent plain chest high-definition computed tomography (HDCT) scans with an HDCT 750 scanner (GE Healthcare, Milwaukee, WI). The scanning method was as follows: after deep inhalation, the patient lay flat without breathing and was scanned from the tip of the lung to the level of the diaphragm. The scanning conditions were as follows: tube voltage 120 kV, tube current 200 mA/s, pitch 0.38, layer thickness 0.625 mm and reconstruction interval 0.625 mm.

### Development of the clinical prediction model

Differences in clinical factors (including clinical data and CT features) between pulmonary CE and pulmonary abscess in the training set were determined using univariate analysis. Multiple logistic regression analysis was used to build a clinical prediction model from the variables identified as significant in the univariate analysis.

### Image segmentation

The volume of a region of interest (ROI**)** at the lesion site was manually described by radiologists with 3 years of experience in chest imaging, and the delineation was repeated twice. Then, the delineation results were confirmed by a senior imaging physician (with more than 10 years of experience in chest diagnosis). Without knowledge of the clinicopathological data, the volume of interest (VOI**)** was defined by tracing along the edge of the lesion. If there was atelectasis and inflammatory exudation around the lesion, a margin of approximately 3–5 mm around the lesion was also traced.

### Radiomics feature extraction

Using RadCloud software (Huiying Medical Technology Co., Ltd), 1409 imaging features were extracted from the VOIs. We used intraclass correlation coefficients (ICCs) to assess the intraobserver and interobserver reproducibility of feature extraction. Initially, we randomly chose 30 VOIs from each modality. The intraobserver ICC was calculated by comparing the 2 segmentations of reader 2 (repeated at a 7-day interval). The interobserver ICC was calculated by comparing the segmentations of reader 1 and reader 2 (first time). When the ICC exceeded 0.8, the agreement was considered good.

### Development of the radiomics signature

To avoid the curse of dimensionality and reduce the bias from radiomics features in the model, dimensionality reduction of the features was performed before signature construction. Briefly, the radiomics features that met the criteria of inter- and intraobserver ICCs greater than 0.8 were tested by one-way analysis of variance (ANOVA) to select potentially important features for the training set. Features that were not significant in either of the above tests were excluded. The remaining features were then included in a least absolute shrinkage and selection operator (LASSO) regression model to select the most valuable features in the training set. LASSO regression has been shown to be effective in reducing overfitting and improving prediction accuracy (Alhamzawi and Ali 2018). The method shrinks the coefficients of many features to zero, retaining those features with nonzero coefficients (Wang et al. 2020). In this study, one standard error of the minimum criteria (the 1-SE criteria, a simple model) was used to tune the regularization parameter (α) in the feature selection using fivefold cross-validation. The selected features were used to build a radiomics signature. A radiomics score (Rad-score) was calculated for each patient via a linear combination of the selected features weighted by their respective LASSO coefficients. Finally, a logistic regression (LR) machine learning model was established by using the reduced dimension features. Accuracy, specificity, sensitivity and the area under the receiver operating characteristic curve (AUC) were used to estimate the predictive performance of the radiomics models. Details and data of the radiomics features are provided in Supplementary Table 1.

### Development of a radiomics nomogram and assessment of the performance of different models

We constructed a radiomics nomogram by incorporating important variables of clinical factors and the Rad-score. A calibration curve was used to evaluate the performance characteristics of the radiomics nomogram. The AUC for the training set and validation set was used to evaluate the diagnostic performance of the clinical factor model, radiologic characteristics and radiologic nomogram. A model with perfect discriminatory ability will have an AUC of 1.0, while a model unable to distinguish between individuals with and without the chosen outcome will have an AUC of 0.50 (Linden. 2010). A radiomics nomogram score (nomoscore) was calculated for each patient in the training and test sets. To evaluate the clinical usefulness of the nomogram, decision curve analysis (DCA) was carried out by calculating the net benefit of a series of threshold probabilities in the whole cohort.

### Statistical analysis

Python 3.6 was used for standardization, feature selection and model building. SPSS 21.0 was used for statistical analysis of the clinical information. First, measurement data were tested for normality. If the data had a normal distribution, the independent sample *t* test was used; if the data did not have a normal distribution, the Mann–Whitney *U* test was used. The chi-square test was used to compare counting data. All statistical tests were two-sided, and *P* values of < 0.05 indicated statistical significance. LASSO regression analysis was performed using the “glmnet” package. Multivariate logistic regression, nomogram calculation and calibration curves were generated using the “rms” package.

## Results

### Patient characteristics

Table 1 shows the clinical characteristics of the patients. Our study included 53 patients with pulmonary CE and 64 patients with pulmonary abscess. Considering *P* < 0.05 to indicate statistical significance, the following clinical indexes were found to be significant: age, monocyte count, IL-6 level, history of hydatid disease, HS-CRP level, density, size, D-dimer level, eosinophil count and temperature.

**Table 1:** Clinical factors of the patients

Variables		Pulmonary CE (*n*=53)	Pulmonary abscess (*n*=64)	*P-value*
Age (Mean ±SD)		35.85 ± 20.47	41.84 ± 20.75	<0.0001*
Gender	Male (%)	37 (69.81%)	37 (57.81%)	0.2512
	Female (%)	16 (30.19%)	27 (42.19%)	
Lesion location	Upper lobe of right lung	18	13	0.1331
	Lower lobe of right lung	12	20	
	Upper lobe of left lung	6	8	
	Lower lobe of left lung	10	20	
	Middle lobe of right lung	7	3	
Sputum	Without	24	36	0.4881
	Yellow	7	4	
	White	20	21	
	Other	2	3	
MOV	Heighten	20	37	0.048*
	Normal	33	27	
IL-6	Heighten	13	38	0.0003*
	Normal	40	26	
FIB	Heighten	20	30	0.074
	Normal	33	34	
NEVT	Heighten	19	31	0.109
	Normal	34	33	
HD-history	Without	44	64	0.0021*
	Have	9	0	
WBC	Heighten	25	30	0.014*
	Normal	28	34	
LYM	Heighten	7	6	0.033*
	Normal	46	58	
HS-CPR	Heighten	16	35	0.0134*
	Normal	37	29	
Density		19.30 ± 11.72	23.73 ± 10.76	<0.0001*
Size		63.82 ± 32.83	61.81 ± 26.58	<0.0001*
D-dimer	Heighten	7	26	0.0021*
	Normal	46	38	
EON	Heighten	16	0	<0.0001*
	Normal	37	64	
Temperature		37.19 ± 1.09	37.79 ± 1.25	<0.0001*
*MOV* monocyte, *NEVT* neutrophils, *HD-history* hydatid-history, *LYM *lymphocyte, *EON* eosnophils				
*P*<0.05 indicated significant differences				

### Radiomics feature extraction and selection and development of the radiomics signature

**Fig. 3:**
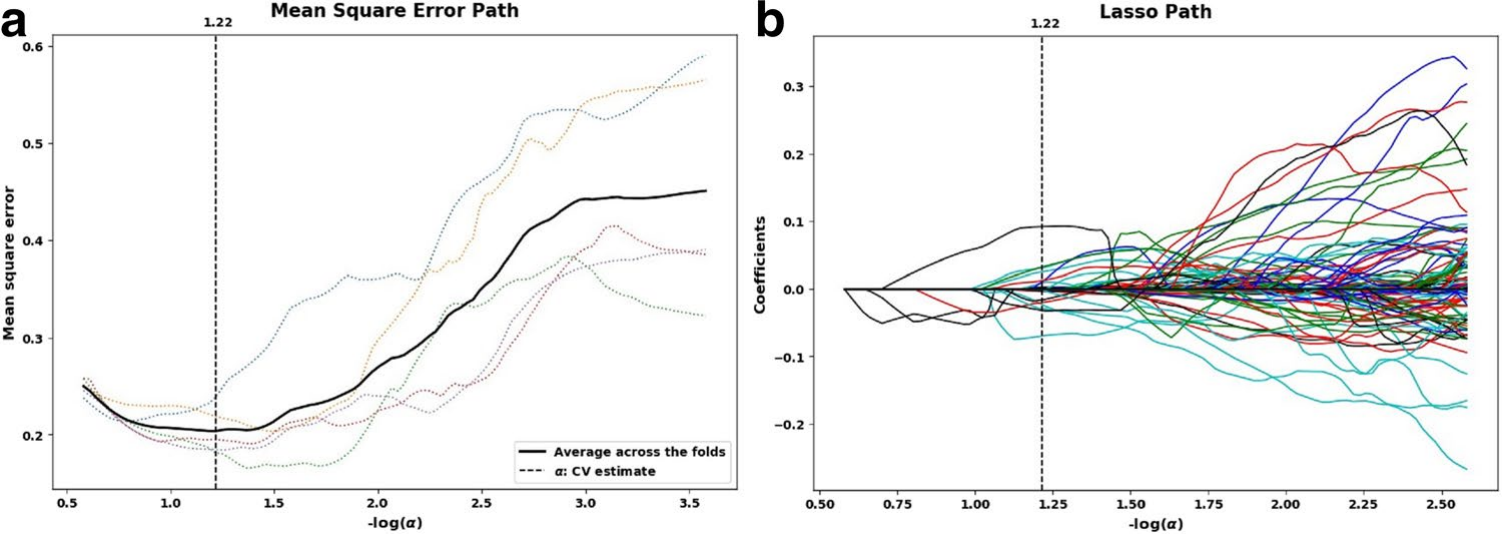
**a** Coefficient distribution map with all the possible values of radiation characteristics. **b** LASSO coefficient profiles of the 1200 radiomics features. A coefficient profile plot was generated versus the selected log (α) value using fivefold cross-validation; the vertical line was plotted with 25 selected radiomics features

Among the 1409 radiological features, the average ICC was 0.9126, and 1200 features had an ICC greater than 0.80 (85.17%), which demonstrates that they have good consistency. The results of the analyses for inter- and intraobserver agreement are provided in Supplementary Table 2; the minimum value was 0.8339, and the maximum value was 1. Using LASSO to screen features, first, the minimum error of each fold was calculated by using the 50% cross-validation method, and an optimal α = 1.22 was obtained (Fig. 3a). Then, values with nonzero feature coefficients were selected from the LASSO analysis (Fig. 3b); that is, the most relevant features for distinguishing pulmonary CE from pulmonary abscess were found, and 25 optimal features were identified, including 18 texture features, 6 first-order features and 1 shape feature. More detailed information about the features can be found in Supplementary Table 3.

### Performance evaluation of individual radiomics features

The performance of the LR machine learning model in terms of the AUC, sensitivity, specificity, recall rate, F1 score and accuracy was compared between the training set and the test set. The AUC of the training group was 0.905 and that of the test set was 0.850. See Table 2 for specific data. Overall, the LR model had good prediction performance.

**Table 2:** Prediction performance of the LR model

	LR training set	LR test set
Cutoff	0.452	0.706
Recall	0.967	0.7
Precision	0.813	1
Sensitivity	0.907	0.7
Specificity	0.827	1
Accuracy	0.863	0.864
F1	0.857	0.824
Brier	0.156	0.174
AUC	0.905	0.858
95% AUC	0.827–0.955	0.644–0.969

### Development of a radiomics nomogram and assessment of the performance of the different models

**Fig. 4:**
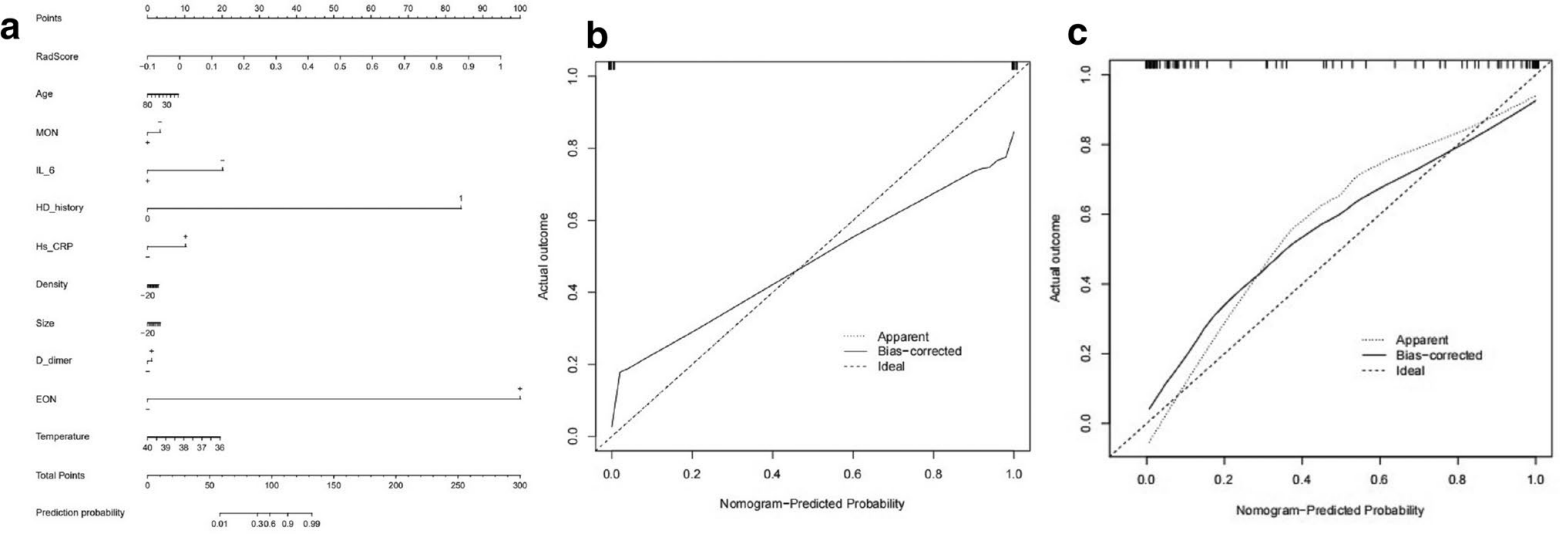
(**a**) Radiomics nomogram and calibration curves for the radiomics features. The radiomics nomogram, combining clinical indicators and the Rad-score, was developed with the training set. Calibration curves for the radiomics nomogram in the test (**b**) and training (**c**) sets, indicating the goodness-of-fit of the nomogram. The 45° straight line represents the perfect match between the actual(Y-axis)and nomogram-predicted(X-axis)probabilities. A closer distance between two curves indicates higher accurary

Combining the Rad-score calculated from the imaging characteristics and 10 different clinical indexes, a comprehensive prediction model and nomogram were constructed (Fig. 4a). The nomogram illustrates that the Rad-score, history of hydatid disease and eosinophil count had the greatest contribution to prediction performance. The calibration curve indicated that the nomogram had good calibration in the training and test sets (Fig. 4b, c).

Table 3 summarizes the AUC value, standard error, 95% confidence interval, sensitivity and specificity of the comprehensive model and shows that the performance of the comprehensive model is obviously better than that of the radiomics model alone.

**Table 3:** Summary of the diagnostic performance of the comprehensive model with the training set and the test set

Parameter	Training set	Test set
AUC	0.97	0.983
Standard error	0.0142	0.0201
95% confidence interval	0.912–0.994	0.816–1.000
Youden index	0.8654	0.9
Sensibility	100	90
Specificity	86.54	100

### Performance of different models

**Fig. 5:**
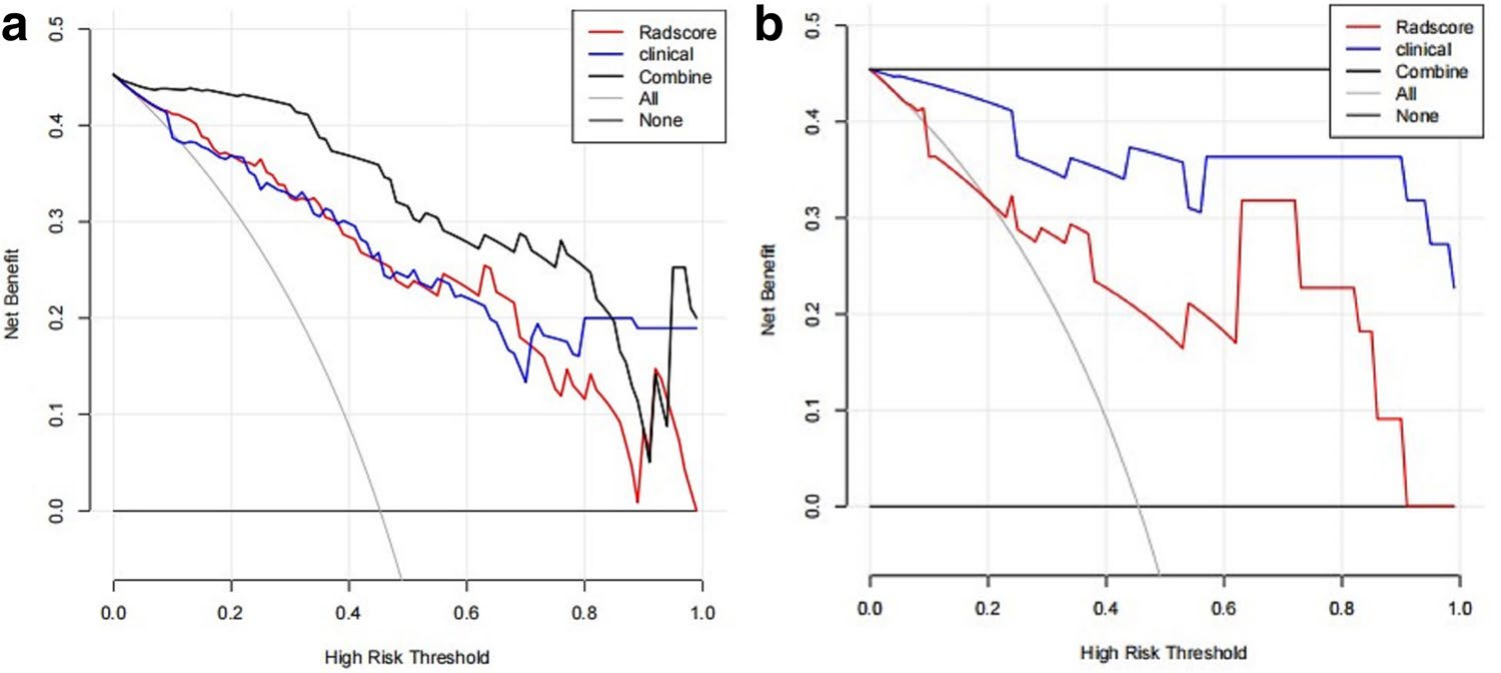
Decision curve analysis of the training set (**a**) and test set (**b**) for the three models. The net benefit is plotted versus the threshold probability. The grey curve represents the assumption that all patients are pulmonary CE patients, and the black curve represents the assumption that all patients have pulmonary abscesses. The red curve represents the radiomics model. The blue curve represents the radiomics nomogram. The black bold curve represents the comprehensive model. The *x*-axis shows the threshold probability. The *y*-axis shows the net benefit. Compared with the other two models and simple diagnosis, the radiomics nomogram has the highest net benefit

DCA (Fig. 5a, b) showed that for most ranges of reasonable threshold probability, in the training set and test set, there was little difference in efficacy between the individual omics and clinical models. The radiomics nomogram had a higher overall net benefit than the clinical prediction model and the single radiomics feature nomogram model in distinguishing pulmonary CE from pulmonary abscess.

## Discussion

The distinction between pulmonary CE and pulmonary abscess is extremely important clinically because the treatment and prognosis of these conditions differ considerably. Xinjiang has one of the highest incidences of CE worldwide. *E. granulosus* was previously considered a single species but is now thought to have extensive genetic diversity, and 10 genotypes (G1-10) have been identified by mitochondrial DNA (mtDNA) sequencing. Recently, it has also been divided into 5 types according to its genetic variation, including *E. granulosus* sensu stricto, *Echinococcus felidis*, *Echinococcus equinus*, *Echinococcus ortleppi* and *Echinococcus canadensis* (Ali et al. 2020; Casulli et al. 2019; Wen et al. 2019). The G1 genotype of *E. granulosus* sensu stricto is overwhelmingly responsible for the global burden of human CE, and infections are widely distributed throughout the world; additionally, this genotype appears to be the sole cause of human CE in many regions with a high prevalence of the condition (Alvarez Rojas et al. 2014; Guo et al. 2019). Studies have shown that G1 is the main epidemic strain in northern Xinjiang (Guo et al. 2019). Generally, the clinical diagnosis of pulmonary CE can be made by epidemiological history, clinical symptoms, imaging findings and serological examination. However, some of the imaging features of ruptured pulmonary CE overlap with those of pulmonary abscess, which makes differential diagnosis with conventional imaging quite difficult (Giri and Parija 2012). This study showed that the AUCs of the training set and test set were 0.905 and 0.858, respectively, when differentiating pulmonary CE and pulmonary abscess with radiology findings alone. The radiomics-based nomogram in the present study showed higher AUCs of 0.970 and 0.983 in the training and test cohorts, respectively, and a higher accuracy of 84% in both cohorts, indicating that it had better performance in distinguishing pulmonary CE from pulmonary abscess. This study showed that adequate clinical and imaging information can support the differential diagnosis of pulmonary CE and pulmonary abscess and is helpful for correctly diagnosing pulmonary CE. In the present study, we identified 10 clinical indexes, among which the eosinophil count and history of hydatid were obviously important. We found that the eosinophil count and history of hydatid in pulmonary CE patients were more obvious than those in pulmonary abscess patients (Zait and Hamrioui 2020; Ravis et al. 2021).

After dimensionality reduction with the LASSO algorithm, 25 optimal features were selected, among which the two features with the greatest correlation were grey level variance (GLV) after wavelet filtering and root mean squared (RMS) after exponential filtering. GLV describes the variance in greyscale values and the size of the greyscale difference in lesions, and RMS is used to describe the intensity of lesions. The arithmetic mean GLV in pulmonary CE was 0.153, and the median value was 0.177; the arithmetic mean GLV in pulmonary abscess was 0.075, and the median value was 1.367. These findings suggest that the grey variance and grey difference in pulmonary CE lesions are significantly higher than those in pulmonary abscesses. The arithmetic mean RMS in pulmonary CE was 1.370, and the median value was 1.367, while those in pulmonary abscess were 1.559 and 1.551, respectively. Therefore, the focus intensity in pulmonary abscess is slightly higher than that in pulmonary CE. Based on the above analysis, grey level changes and lesion intensity may be helpful for the differential diagnosis of pulmonary CE and pulmonary abscess, but the data of this study are not sufficient. In the future, the sample size and number of clinical characteristics should be increased.

Radiology has been used widely in the differential diagnosis of chest conditions. Previous studies have shown that nomograms can be used to distinguish between renal angiomyolipoma without visible fat (AML.wovf) and homogeneous clear cell renal cell carcinoma (hm-ccRCC), with AUCs of 0.896 and 0.949 in the training group and verification group, respectively, indicating good value in distinguishing the two conditions (Nie et al. 2020). Another study used clinical data and radiological features extracted from chest CT images to develop a model for predicting the survival and prognosis of coronavirus disease 2019 (COVID-19) patients (Shiri et al. 2021). Recently, some studies combined chest CT radiograms and clinical features into a COVID-19 risk score to distinguish COVID-19 from other viral pneumonias to better achieve early diagnosis and treatment of the former (Chen et al. 2021). Radiology has also been applied in the differential diagnosis of pulmonary tuberculosis (TB) and lung cancer (LC). The nomogram in that study showed good discriminability, and the model had good sensitivity and specificity, greatly improving the clinical diagnosis of TB and LC (Cui et al. 2020). This study also applied a nomogram combining radiologic labels and clinical indicators. There were 10 clinical indicators and 25 radiologic features that were meaningful for the comprehensive model in this study and were especially meaningful for the differential diagnosis of pulmonary CE and pulmonary abscess.

Our research has several limitations. First, there are a large number of mutated strains and polymorphic genes in the *Echinococcus* genus, resulting in different levels of infectiousness to different types of hosts in different geographical environments, and their antigen specificity, pathogenicity and transmission ability are also different. This study is a single-centre, small-sample retrospective study. Even if the amount of data is repeatedly calculated and validated, the amount of data and the scope are small; this lack of a large number of samples and polygenotypes of echinococcosis can lead to uncertainty. Second, the segmentation of pulmonary CE and pulmonary abscess has not been standardized. Manual segmentation usually leads to changes in the lesion boundary due to differences between observers. It should be noted that in its current state, radiomics cannot completely replace the manual work of radiologists or tissue examination. More effort is needed to overcome the limitations identified above to facilitate the widespread application of radiomics in the near future.

## Conclusion

The radiomics nomogram based on the radiomics signature and clinical factors developed in this research performed well in distinguishing pulmonary CE and pulmonary abscess and has great potential in clinical diagnosis.

## Supplementary Information

Below is the link to the electronic supplementary material.

Supplementary file1 (XLS 66 KB)

Supplementary file2 (XLS 31 KB)

Supplementary file3 (XLS 191 KB)

Supplementary file4 (XLS 45 KB)

Supplementary file5 (XLS 22 KB)
